# Supplementary material for: Graph Theory-Based Electroencephalographic Connectivity and Its Association with Ketogenic Diet Effectiveness in Epileptic Children
Source: Nutrients. 2021 Jun 25;13(7):2186. doi: 10.3390/nu13072186 (PMC8308392; doi:10.3390/nu13072186)
Supplement: Supplementary file 1 [file nutrients-13-02186-s001.zip › nutrients-1217010-supplementary.pdf]

nutrients

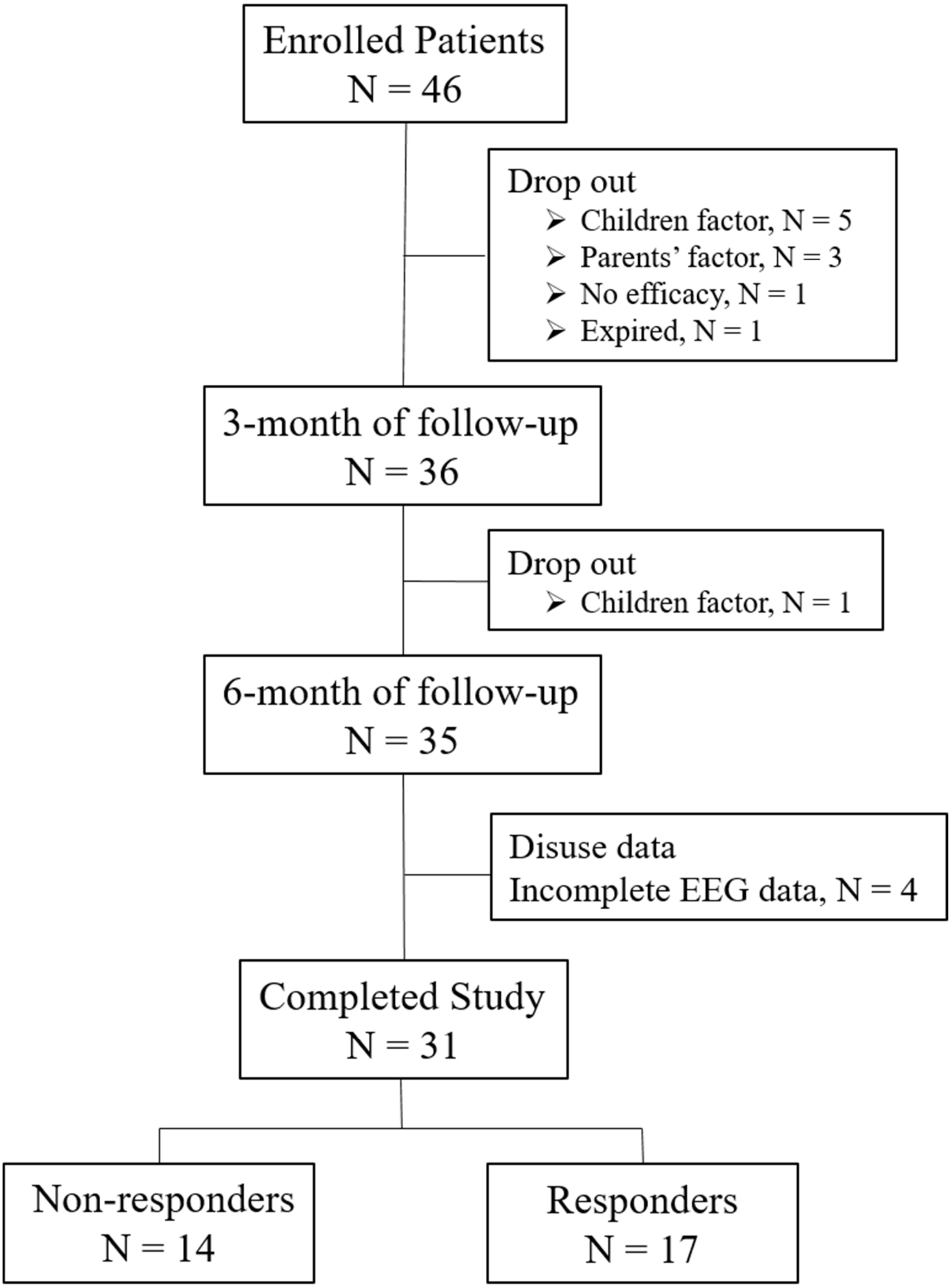

Figure S1. Flowchart of patient enrollment. We enrolled 46 subjects initially. Ten of them dropped out before 3-month follow-up, and one before 6-month follow-up due to children's factors, parents' factors, poor efficacy, or death. Four subjects were excluded for incomplete EEG data. Thirty-one subjects were finally included in this study, of whom 17 were KDT responders and 14 were non-responders.

Table S1. Patients' document. The clinical raw data of responders and non-responders were listed in this table. The six months seizure frequency means the average frequency during the first 6 months of ketogenic diet therapy. The six months seizure reduction rate (%) = (Baseline seizure frequency –six months seizure frequency) / Baseline seizure frequency x 100%. Responders were those who achieved a six months seizure reduction rate of 50% or more.

Age (Y; M)	Gender	Main Seizure type (ILAE 2017)	Etiology	Baseline Number of AEDs	Baseline Seizure Frequency (per month)	Six months Seizure Frequency (per month)	Six months Seizure Reduction rate (%)
**13; 6**	F	Focal to bilateral tonic-clonic	genetic	1	1	0	100.00
**0; 9**	M	Generalized onset, Motor	HIE	2	5	0	96.67
**2; 4**	M	Generalized onset, Motor	HIE	4	476	145	69.64
**15; 4**	F	1. Focal onset, Impaired awareness, Non-motor onset 2. Focal to bilateral tonic-clonic	unknown	4	1	0	100.00
**6; 10**	F	Focal onset, Impaired awareness, Non-motor onset	genetic	2	15	0	98.89
**2; 6**	M	Generalized onset, Motor	HIE	3	252	31	87.63
**10; 3**	M	Focal to bilateral tonic-clonic	unknown	3	224	33	85.12
**3; 0**	M	Generalized onset, Motor	genetic	3	224	19	91.59
**1; 2**	M	Generalized onset, Motor	structural	3	168	28	83.23
**2; 4**	F	1. Generalized onset, Motor 2. Focal to bilateral tonic-clonic	genetic	2	8	4	50.00
**13; 10**	F	Generalized onset, Motor	genetic	2	4	1	75.00
**0; 10**	F	1. Focal to bilateral tonic-clonic 2. Focal onset, Impaired awareness, Non-motor onset	genetic	4	3	1	77.78
**4; 11**	F	Generalized onset, Motor	unknown	3	196	88	55.10
**15; 1**	F	Focal to bilateral tonic-clonic	unknown	2	1	0	66.67
**13; 10**	F	Focal onset, Impaired awareness, Non-motor onset	genetic	2	6	2	72.22
**4; 4**	M	1. Focal onset, Impaired awareness, Non-motor onset 2. Focal onset, Impaired awareness, Motor onset	unknown	2	9	2	77.78
**2; 11**	M	Generalized onset, Motor	HIE	5	168	0	100.00
**19; 1**	M	Generalized onset, Motor	HIE	2	1	2	-83.33
**14; 4**	M	1. Focal onset, Impaired awareness, Motor onset 2. Focal to bilateral tonic-clonic	immune	3	33	19	43.94
**10; 10**	M	Generalized onset, Motor	unknown	2	1	1	-33.33

**13; 2**	F	Generalized onset, Motor	unknown	2	280	222	20.83
**10; 0**	M	1. Generalized onset, Nonmotor<br>2. Generalized onset, Motor<br>3. Focal to bilateral tonic-clonic	genetic	2	32	18	44.79
**1; 2**	M	Generalized onset, Motor	genetic	2	70	37	46.90
**0; 10**	F	1. Focal onset, Impaired awareness, Motor<br>2. Focal to bilateral tonic-clonic	structural	4	30	80	-165.00
**9; 7**	F	Focal onset, Impaired awareness, Non-motor	infectious	3	1	2	-116.67
**10; 4**	F	Focal onset, Impaired awareness, Non-motor	genetic	3	66	58	12.37
**0; 3**	F	Generalized onset, Motor	structural	3	51	33	34.64
**0; 3**	F	Generalized onset, Motor	genetic	4	41	48	-17.07
**6; 0**	M	Focal to bilateral tonic-clonic	genetic	1	5	11	-123.33
**6; 1**	F	Generalized onset, Motor	genetic	6	140	200	-0.429
**0; 9**	M	Generalized onset, Motor	genetic	3	140	100	0.286

Y: year; M: month; F: Female; M: Male; ILAE: International League Against Epilepsy; AED: anti-epileptic drug; HIE: hypoxic ischemic encephalopathy.
